# *Pachybrachis sassii*, a new species from the Mediterranean Giglio Island (Italy) (Coleoptera, Chrysomelidae, Cryptocephalinae)

**DOI:** 10.3897/zookeys.155.1951

**Published:** 2011-12-15

**Authors:** Matteo Montagna

**Affiliations:** 1 DIPAV, Sezione di Patologia Generale e Parassitologia, Facoltà di Medicina Veterinaria, Università degli Studi di Milano, Italy; 2 DIPSA, Dipartimento di Protezione dei Sistemi Agroalimentare e Urbano e Valorizzazione della Biodiversità, Facoltà di Agraria Università degli Studi di Milano, Italy

**Keywords:** Entomology, taxonomy, Coleoptera, Chrysomelidae, Cryptocephalinae, Pachybrachini, Tuscan Archipelago, neotype

## Abstract

*Pachybrachis sassii*, new species is described from Giglio Island, of the Tuscan Archipelago (Italy). The new species belongs to the nominotypical subgenus and is closely related to *Pachybrachis salfii* Burlini, 1957, from which it differs in the shape of the median lobe of the aedeagus and in the pattern of the yellow raised spots on the elytra and pronotum. Ecological observations are made. The neotype of *Pachybrachis salfii* from Colloreto, Monte Pollino (Italy) is designated.

## Introduction

The genus *Pachybrachis* Dejean, 1836 belongs to the subfamily Cryptocephalinae (Coleoptera, Chrysomelidae) and according to the color of the prothorax and elytra is subdivided into two subgenera: *Pachybrachis* sensu stricto (hereafter s. str.) and *Chloropachys* Rey, 1883. Phylogenetic analyses based on mitochondrial (12S rRNA, 16S rRNA) and nuclear markers (18S rRNA and 28S rRNA) (Hsiao 1994, Gomez-Zurita et al. 2007) confirmed the basal position of *Pachybrachis* within the Cryptocephalinae clade.

More than 350 species of *Pachybrachis* s. str. have been described till now, about 200 of which are distributed in the western hemisphere and 150 in the Palaearctic Region (Warchalowski 2008). In the last decades about 45 new species of *Pachybrachis* s. str. have been described from the Palaearctic Region. Nowadays, 51 species are listed in the European fauna, 23 species are present in Italy (Sassi 2004, 2005). Interestingly, in the Mediterranean basin *Pachybrachis* s. str. shows a high level (19.6%) of island endemism. *Pachybrachis* is a polyphagous genus, its species are feeding on plants belonging to different families (Jolivet and Hawkeswood 1995; Bieńkowski 1999).

In the summer of 2010, during study of a Chrysomelidae fauna of the Mediterranean islands, 11 specimens of a previously unknown *Pachybrachis* were collected. The specimens appeared closely related to *Pachybrachis salfii* Burlini, 1957 from which they differed in the structure of the median lobe of the aedeagus and in the pattern of yellow raised spots of the elytra and pronotum. Genital characters together with a pattern of yellow raised spots are commonly used for discrimination of sibling species within this genus (Burlini 1968, Sassi and Schöller 2003).

## Material and methods

All the specimens were collected on the host plants by net sweep. Specimen manipulation, dissection, measure, and photographs were completed with the auxiliary use of the stereo microscope Leica MS5 with an ocular micrometer. SEM micrographs of the aedeagus were made using Jeol JSM-5610LV scanning electron microscope. Spermatheca and kotpresse drawings were made using stereomicroscope Leica MS5 with an ocular grid (size 20 × 20 squares 1 cm²). Specimens are deposited at the Civic Museum of Natural History, Milan (MSNM), Matteo Montagna private collection, Anzano del Parco, Como, Italy (MMPC), and Davide Sassi private collection, Castelmarte, Como, Italy (DSPC).

### Key to *Pachybrachis* s. str. with well defined yellow spots or/and stripes on a black background similar to *Pachybrachis sassii* sp. n. distributed in Italy

Due to a complexity of the genus (Schöller 2010), the present key is not based on a phylogenetic hypothesis. It includes five species that are most similar to *Pachybrachis sassii*by the black and yellow pattern of the pronotum and elytra.

**Table:** 

1	Mesoepimeron black, sometimes with blurred indistinct yellow shadow	2
–	Mesoepimeron yellow	4
2	Last abdominal tergite entirely black, without yellow spots. Mid and hind femora fulvous with lighter spot. Length: 3–3.5 mm. Distribution: Central Europe, Spain, France and Italy	*Pachybrachis picus* Weise, 1882
–	Last abdominal tergite with yellow spots	3
3	Last abdominal sternite black, without spots. Black spot on pronotum with lateral yellowish dots. Length 3.5–4 mm. Distribution: South Central Europe, from Spain to the Caspian Sea	*Pachybrachis tesselatus* Olivier, 1791
–	Last abdominal sternite with two yellow spots. Black spot on pronotum without lateral yellowish dots. Length: 3.5–4 mm. Distribution: Spain, France, Italy, Croatia, Serbia and Montenegro	*Pachybrachis exclusus* Rey, 1883
4	Elytron without convex yellow spots or stripes elevated over the background	5
–	Elytron with convex yellow spots or stripes elevated over the background. Mesoepimeron with yellow sometimes indistinct spot. Last abdominal tergite and sternite completely black. Length 2.8–3.4 mm. Distribution: Giglio island (Italy)	*Pachybrachis sassii* sp. n.
5	Last abdominal sternite without two yellow spots. Length: 3.1–4.2 mm. Distribution: Europe and West Siberia	*Pachybrachis hieroglyphicus* Laicharting, 1781
–	Last abdominal sternite with two yellow spots. Length: 2.8–3 mm. Distribution: Sicily	*Pachybrachis siculus* Weise, 1891

## Taxonomy

### Pachybrachis (Pachybrachis) sassii

Montagna,  sp. n.

urn:lsid:zoobank.org:act:4AD3247F-94F2-43F3-8F89-FFD6D2F3B4FB

http://species-id.net/wiki/Pachybrachis_sassii

Figs 1 3 6 

#### Type-locality.

Italy, Tuscany: Grosseto, Giglio island, Giglio Campese.

#### Material examined.

Type-specimens:Holotype male, pinned, with genitalia on a separate card board. Original label: “Italy, Grosseto, Isola del Giglio, Giglio Campese, 42°21.90'N, 10°52.59'E, ca. 30 m a.s.l., 14 Jun 2010, M. Montagna & F. Castiglioni leg. [printed label], “HOLOTYPUS / *Pachybrachis sassii* / Montagna M. des. [red handwritten label] (MSNM). Paratypes: 5 females and 5 males pinned. Three females and 3 males, original label: “Italy, Grosseto, Isola del Giglio, Via della Cabulgina, 42°22.97'N, 10°53.89'E, ca. 60 m a.s.l., 13 Jun 2010, M. Montagna & F. Castiglioni leg.” [printed label], “PARATYPE / *Pachybrachis sassii* / Montagna M. des. [red handwritten label]; 2 males and 2 females (MMPC), 1 male and 1 female (DSPC). Two males and 2 females, original label: “Italy, Grosseto, Isola del Giglio, Giglio Campese, 42°21.01'N, 10°52.26'E, ca. 30 m a.s.l., 14 Jun 2010, M. Montagna and F. Castiglioni leg. [printed label], “PARATYPE / *Pachybrachis sassii* / Montagna M. des. [red handwritten label] (MMPC).

#### Diagnosis.

The new species belongs to the subgenus *Pachybrachis* s.str. The external morphological characters, general habitus, size, black and yellow pattern of elytra and pronotum prompt the ascription of *Pachybrachis sassii* sp. n. to the fifth artificial group suggested by Burlini (1968) and adopted in the most recent revision of the subgenus by Warchalowsky (2008). Species belonging to this group are longer than 3 mm, have black elytra with yellow raised spots, mesoepimera completely black or sometimes with an indistinct yellow spot, pronotum with irregular and deep punctation.

#### Description.

Male and female measurements are reported in Table 1.

**Figures 1–2.**
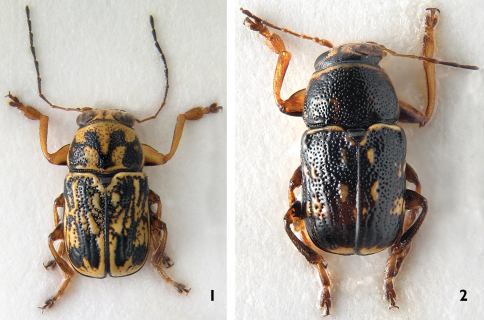
Habitus, dorsal view: **1** *Pachybrachis sassii* sp. n., holotype **2** *Pachybrachis salfii*, neotype.

Body relatively robust, dorsally black with yellow raised spots (Fig. 1), black/yellow ratio variable in male and female (female usually darker than male). Ventral side black, mesoepimera with yellow sometimes evanescent spot. Pygidium black. Vertex, frons, clypeus and upper labrum yellow. Antennal insertions, median longitudinal line on frons (in female wider and triangular in shape) and upper internal margin of eyes black. Vertex with black transverse line. First three antennomeres pale with blackish upper part, from IV to XI blackish to completely black. Pronotum with black/yellow pattern and narrow blackish outline, entirely black in some female. Two large lateral and irregular black spots reach pronotal base at corners, sometimes contact with anterior border in female. Lateral and anterior edges yellow, absent in some female. Evanescent yellow mark present within lateral black spot. Central black “V”-shaped spot connected to lateral spots. Scutellum black, shiny. Elytron with yellow shiny edge extending from humeral callus to suture surrounding scutellum. Humeral callus shiny, proximally yellow, distally black, completely black in some specimens. Suture black. Epipleura yellow with black outline. Apical lunula yellow, covered with evident black punctures in middle, inner and outer yellow branches extending up to half of suture and below humeral calli respectively. Legs brownish, upper part of femora black in male. Mid and hind femora with distinct yellow spots and black knees. Frons flat, interocular furrow fairly impressed and punctured, evanescent in female. Eyes at upper edge with impressed and punctured median line. Head with strong and diffuse punctation, denser in middle and close to internal margins of eyes, evanescent on clypeus. First antennomere swollen, as long as third, second antennomere roundish and shorter than half of third. Eyes prominent, distance between upper lobes smaller than distance between antennal sockets.

**Figures 3–4.**
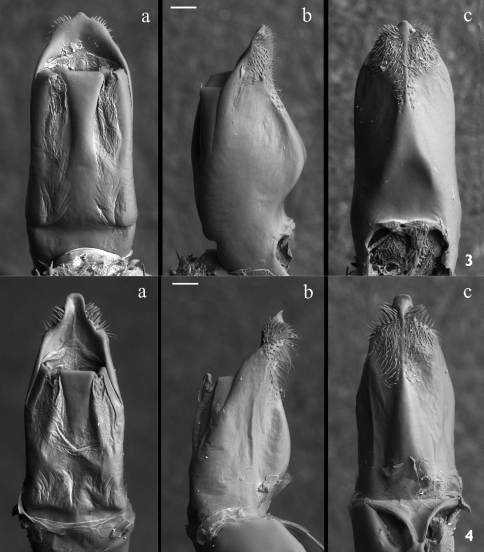
Median lobe of aedeagus; magnification 120×, scale bar = 100 µm **3** *Pachybrachis sassii* sp. n. holotype; **a** dorsal **b** lateral and **c** ventral view **4** *Pachybrachis salfii* neotype **a** dorsal **b** lateral and **c** ventral view. Median lobe silhouette is demaged by dehydration, probably due to contact with air for a long time.

**Figure 5.**
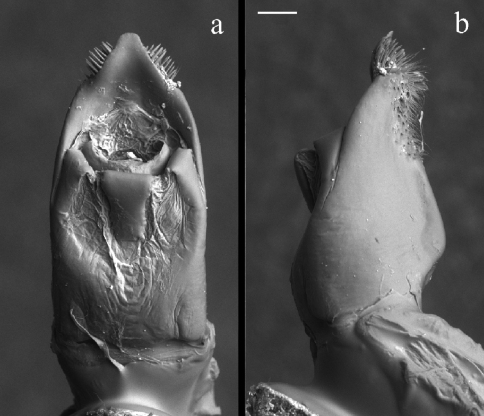
Median lobe of aedeagus: *Pachybrachis salfii* from Monte Pollino freshly extracted **a** dorsal and **b** lateral view. Magnification 120×; scale bar = 100 µm.

**Figure 6.**
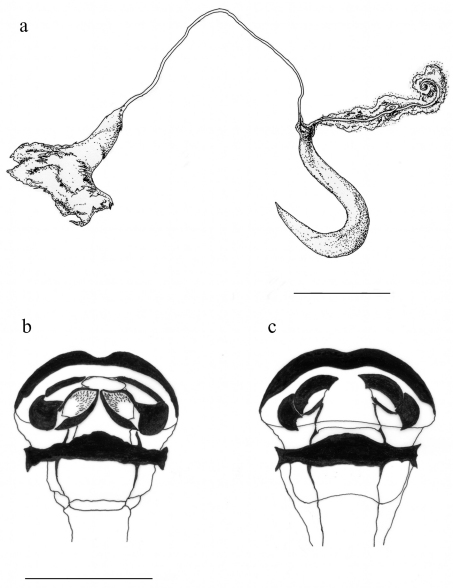
*Pachybrachis sassii* sp. n. **a** spermatheca **b** ventral sclerite of kotpresse **c** dorsal sclerite of kotpresse. Scale bar = 0.3 mm.

Pronotum with maximum width slightly posterior to middle. Longitudinal edge curved in middle, anteriorly not completely visible from above. Posterior edge sinuous in middle, slightly rimmed and bordered by line of punctures. Punctation evanescent on central disc close to median yellow spot, gradually coarser on sides and towards posterior margin, absent on tiny yellow raised ridge surrounding pronotum. Posterior margin of pronotum with single row of punctures. At bottom, two transverse impressions separated from edge by slightly raised area, less evident than in *Pachybrachis salfii*. Scutellum wide, rectangular, slightly convex, covered with short setae. Elytra slightly wider than pronotum at base, flattened on top. Elytral punctation located mainly on black markings, well impressed and placed in irregular rows anteriorly, starting from median spot becomes almost regular and less impressed. Humeral callus prominent, externally delimited by grooves. Base of elytra, proximally to margin, with evident carina originating at humeral calli and extending along anterior margin up to suture surrounding scutellum. Epipleura narrowly raised. Internal margin of apical lunula evenly cut, puncture clusters separate two inner appendices. Pygidium evenly convex, punctated and pubescent. Urosternite with evident microsculpture and fine whitish pubescence. In male external margin of VII urosternite with row of short and dense setae. Anal sternite with shiny median impression in male, rectangular and not deeply engraved fovea in female. Legs without diagnostic characters, first tarsomere enlarged in male. Median lobe of aedeagus well sclerotized. Phallotreme with sinuous edges, tiny truncated tip surrounded by visible setae, central frenulum with long basal stem expanded anteriorly (Fig. 3a). In lateral view (Fig. 3b) apex sinuous, not curved apically as in *Pachybrachis salfii* (Figs 4, 5); frenuli narrow with right angle in distal part, not obtuse as in *Pachybrachis salfii* (Figs 4, 5). Ventral side with evident triangular carina harboring two small cristae at base, apex with robust setae on each side and hairless tip (Fig. 3c). Spermatheca (Fig. 6a) sickle-shaped, wider on curve, slender anteriorly to fine tip, slightly recurved at base; ductus sclerotized and smooth throughout its length, at base robust junction with spermatheca, distal end forming a subtriangular pigmented diverticulum; accessory gland joining spermatheca at base in slightly sclerotized junction opposite to ductus. Ventral sclerites of kotpresse (Fig. 6b) triangular, lateral apices with three spicules each, anterior margin with prominent central carina, posterior margin sinuous and convex in middle; dorsal sclerite strongly transverse (Fig. 6c), two narrow lateral wings and two well sclerotized vertical processes.

#### Etimology.

The species is dedicated to Dr. Davide Sassi, a friend and well-known specialist in Chrysomelidae.

#### Ecology.

*Pachybrachis sassii* inhabits plant geoseries of *Cyclamino repandi-Querco ilicis* sigmetum (De Dominicis et al. 2010) that are composed by fragmented bush vegetation close to sea reef. Specimens of *Pachybrachis sassii* were observed and collected on *Erica arborea* Linné and on *Cistus incanus* Linné. The mating male and female were observed on *Cistus incanus*. Adults were active in sunny days from mid-morning until mid-afternoon, when the temperature is the highest.

**Table 1.** Male and female measurements of *Pachybrachis sassii* sp. n.: mean (with 95% confidence interval) in mm, standard deviation (in parentheses), range of variability (in square brackets) in mm. TotL: total length; ProL: length of pronotum; ElyW: width of elytra (measured at the base); ProW: width of pronotum, Ratio L/W (ratio between total body length and width of elytra).

Specimens	TotL	ProL	ElyW	ProW	Ratio L/W
Male	3.12 ± 0.15 (0.19)	0.98 ± 0.04 (0.05)	1.55 ± 0.06 (0.07)	1.42 ± 0.05 (0.07)	1.94–2.12
n = 6	[2.76–3.27]	[0.87–1.02]	[1.42–1.64]	[1.29–1.49]	
Female	3.20 ± 0.10 (0.11)	0.92 ± 0.03 (0.04)	1.63 ± 0.05 (0.05)	1.46 ± 0.03 (0.04)	1.91–1.98
n = 5	[3.05–3.38]	[0.87–0.98]	[1.56–1.71]	[1.45–1.53]	[1.45–1.53]

## Discussion

Morphological characters of the aedeagus highlight the affinity between *Pachybrachis sassii* and *Pachybrachis salfii*, as both taxa show the median lobe with a peculiar triangular carina on the ventral side, nevertheless, they differ in the shape of phallotreme, apical tip and lateral apex profile (Figs 3, 4, 5). The tip of the aedeagus in *Pachybrachis sassii* is sinuous and not curved anteriorly as in Cistus*Pachybrachis salfii*. These two taxa also significantly differ in the black and yellow pattern on pronotum and elytra (Figs 1, 2). Present data does not allow to test different hypotheses for the origin of the new taxon but the pre-Pleistocene transgression that separated Giglio island from Argentario cape could have been the event that lead to the allopatric speciation.

During the present study, the author discovered that the holotype of *Pachybrachis salfii* Burlini, 1957, deposited in the collection of Museo Zoologico dell’Università Federico II di Napoli, is lost (personal communication of Dr. Nicola Maio, curator, October 2010). According to article 75 of the International Code of Zoological Nomenclature (1999) and in order to clarify the taxonomic status of the nominal taxon, a paratype of *Pachybrachis salfii* (Burlini, 1957) is designated as neotype. Dr. Carlo Leonardi and Dr. Davide Sassi were previously consulted in order to avoid the proposed designation arousing serious objections. The neotype of *Pachybrachis salfii*  was designated as paratype by Burlini in 1957. The specimens has 4 labels: 1) Colloreto / VI - 951 [printed]; 2) Mass. Pollino / Ruffo l. [printed]; 3) Paratipo [red label, printed]; 4) *Pachybrachys Salfii* m. ♂ [handwritten by M. Burlini]/ det Burlini 1956 [printed]; disegno / dell’olotipo / di Colloreto, / ora in Museo / di Napoli [handwritten by M. Burlini]; 5) *Pachybrachis salfii* /NEOTYPE/ Montagna M. des. [printed]. The neotype of *Pachybrachis salfii* is preserved in the collections of Civic Museum of Natural History, Verona, Italy. The habitus and aedeagus of the neotype are illustrated (Figs 2, 4).

## Supplementary Material

XML Treatment for Pachybrachis (Pachybrachis) sassii
